# Effect of the physiognomy of *Attalea butyracea* (Arecoideae) on population density and age distribution of *Rhodnius prolixus* (Triatominae)

**DOI:** 10.1186/s13071-015-0813-6

**Published:** 2015-04-01

**Authors:** Plutarco Urbano, Cristina Poveda, Jorge Molina

**Affiliations:** Centro de Investigaciones en Microbiología y Parasitología Tropical - CIMPAT, Universidad de los Andes, Carrera 1 No. 18A-10, Bloque A, Bogotá, Colombia

**Keywords:** Population density, *Trypanosoma cruzi*, *Rhodnius prolixus*, Palm trees, Casanare

## Abstract

**Background:**

*Rhodnius prolixus* Stål, 1859 is one of the main vectors of *Trypanosoma* (*Schyzotrypanum*) *cruzi* Chagas, 1909. In its natural forest environment, this triatomine is mainly found in palm tree crowns, where it easily establishes and develops dense populations. The aim of this study was to evaluate the effect of the physiognomy and reproductive status of *Attalea butyracea* on the population relative density and age structure of *R. prolixus* and to determine the vector’s population stratification according to the vertical and horizontal profile of an *A. butyracea* forest.

**Methods:**

Using live bait traps, 150 individuals of *A. butyracea* with different physiognomy and 40 individuals with similar physiognomy (crown size, number of leaves, palm tree height, diameter at breast height, reproductive status) were sampled for triatomines in Yopal, Casanare-Colombia. Temperature and relative humidity were measured in the crown of the palm tree. Entomological indices and natural infection rates were also determined.

**Results:**

The relative population density of *R. prolixus* on natural *A. butyracea* groves is associated with the palm’s height, number of leaves and crown volume. The young immature stages were present mostly at the crown’s base and the advanced immature stages and adults were present mostly at the crown of the palm tree. This distribution correlates with the temperature stability and relative humidity in the base and the fluctuation of both environmental variables in the palm’s crown. A higher density of *R. prolixus* was found as the palm tree height increased and as the distance of the palm with respect to the forest border decreased, especially towards anthropically intervened areas. A density index of 12.6 individuals per palm tree with an infestation index of 88.9% and a colonization index of 98.7% was observed. 85.2% was the infection index with *T. cruzi*.

**Conclusion:**

The physiognomy of palm trees affects the relative population density and the distribution of developmental stages of *R. prolixus*. Therefore, they constitute a risk factor for the potential migration of infected insects from wild environments towards residential environments and the subsequent epidemiological risk of transmission of *T. cruzi* to people.

## Background

Chagas disease is an anthropozoonosis caused by the parasite *Trypanosoma* (*Schyzotrypanum*) *cruzi* Chagas, 1909 that currently affects approximately 10 million people, with another 25 million at risk for infection [1]. In Colombia, it is estimated that almost 1.3 million people are infected, and nearly 3.5 million inhabit risk areas for *T. cruzi* infection [2].

The main transmission mechanism of this parasite is through the feces of vector insects of several species of the Triatominae subfamily (Hemiptera: Reduviidae) [3]. Some investigations have demonstrated that approximately 72 of the 148 Triatominae species are classified as potential *T. cruzi* vectors [4,5]. In Colombia, 26 triatomine species have been registered [6-8], of which 15 were reported to have natural infections with *T. cruzi* [4]. Among them, *Rhodnius prolixus* Stål, 1859 and *Triatoma dimidiata* (Latreille, 1811) stand out as the main vectors in several regions of the country [9]. Oral outbreaks of Chagas disease have also been reported in Colombia involving sylvatic triatomines and sylvatic genotypes of *T. cruzi* [10,11].

For Chagas disease, two basic vector transmission cycles of *T. cruzi* have been reported. The first is a domestic cycle that is characterized by the intrusion or colonization of triatomines in domestic environments, which enables the transmission of the parasite to humans and domestic animals. The second is a wild cycle, in which the transmission of the parasite occurs between wild vertebrates and triatomines [12] inhabiting altitude ecotopes such as palm trees, animal caves, underground burrows and bird nests [3,13,14].

Among the altitude ecotopes, approximately 20 species of palm trees have been found to be infested with colonies of triatomine species infected with *T. cruzi* [3,7,9,13-21]. The genus *Attalea* H.B.K. 1816 is the most frequently infested palm tree, constituting an important natural ecotope for 18 triatomine species, mainly of the genus *Rhodnius* [3,7,13,16-21]. *Attalea butyracea* (Mutis ex L.f.) Wess. Boer is the most natural biotope found infested by triatomine species in different regions of Colombia [19-25].

These palm trees enable triatomine colonization due to the wide range of available microhabitats with stable microclimates. These conditions are generated by their morphological and biological characteristics [26,27]. The conditions also favor the arrival and establishment of a high number of vertebrate species, which act as food sources for the triatomine populations that are established on the palm tree crown [14]. This fact has allowed ecological adaptations in species like *R. prolixus* for survival in these microhabitats and to establish close associations with wild palm trees [23]. Some studies have demonstrated that this association may present a degree of specificity, which varies from a one-to-one association to several triatomine species sharing the same palm tree [15,24].

The density, age structure and temporal variation in *Rhodnius pallescens* (Barber, 1932) [25] and *Rhodnius robustus* Larrouse, 1927 [28] stand out among the main aspects that have been studied to determine the association between the triatomines and palm tree species. Moreover, the population density, spatial variation and age structure of *R. prolixus* in natural *A. butyracea* forests can also be highlighted [24]. Conversely, it has been shown that the population density of other *Rhodnius* species, such as *Rhodnius ecuadoriensis* (Lent & León, 1958), is negatively affected by the variation of microclimate conditions on the microhabitats offered by the palm tree crowns [29]. This result is because the microclimate conditions of the different niches offered by these plants can vary according to the environmental conditions and the structural characteristics of the palm tree species [30].

The physiognomy of palm trees varies according to the landscape where they are growing and the reproductive biology of each species [31]. The synchrony in the phenophases of reproductive events, the growth habit and the frequency of abscission of dry leaves and reproductive structure debris determine the different structural physiognomies in the palm tree species [32,33]. Moreover, the growth type influences the interspecific interactions with other plants, which favors the biomass accumulation in their crowns [31,32].

*A. butyracea* is a species that, in addition to the above biological characteristics, has reproductive events throughout the year. This characteristic allows the accumulation of dry biomass on its crown for long periods [31-33], favoring the availability of relatively stable microhabitats [34]. This fact has allowed the maintenance and establishment of *R. prolixus* colonies on a space-time scale that also favors the possibility of dispersal towards other ecotopes used by this vector [20,35].

According to the aforementioned studies [30-34], it could be hypothesized that the variation of the structural physiognomy and reproductive status of the *A. butyracea* individuals could affect the density, population micro-distribution and presence of the developmental stages of *R. prolixus* within the microhabitats generated in the crown palm.

To provide information to address the stated hypothesis, this study evaluated the effect of the physiognomy and reproductive status of *A. butyracea* palm trees on the relative population density and age structure of *R. prolixus*. In addition, the vector’s population distribution according to the vertical (base, mid-zone and upper part of the crown of the palm trees) and horizontal (localization of the palm trees in the forest, borders or inside the forest) profile of the forest studied was determined, and some entomological and *T. cruzi* infection indices were calculated.

## Methods

**Figure 1:**
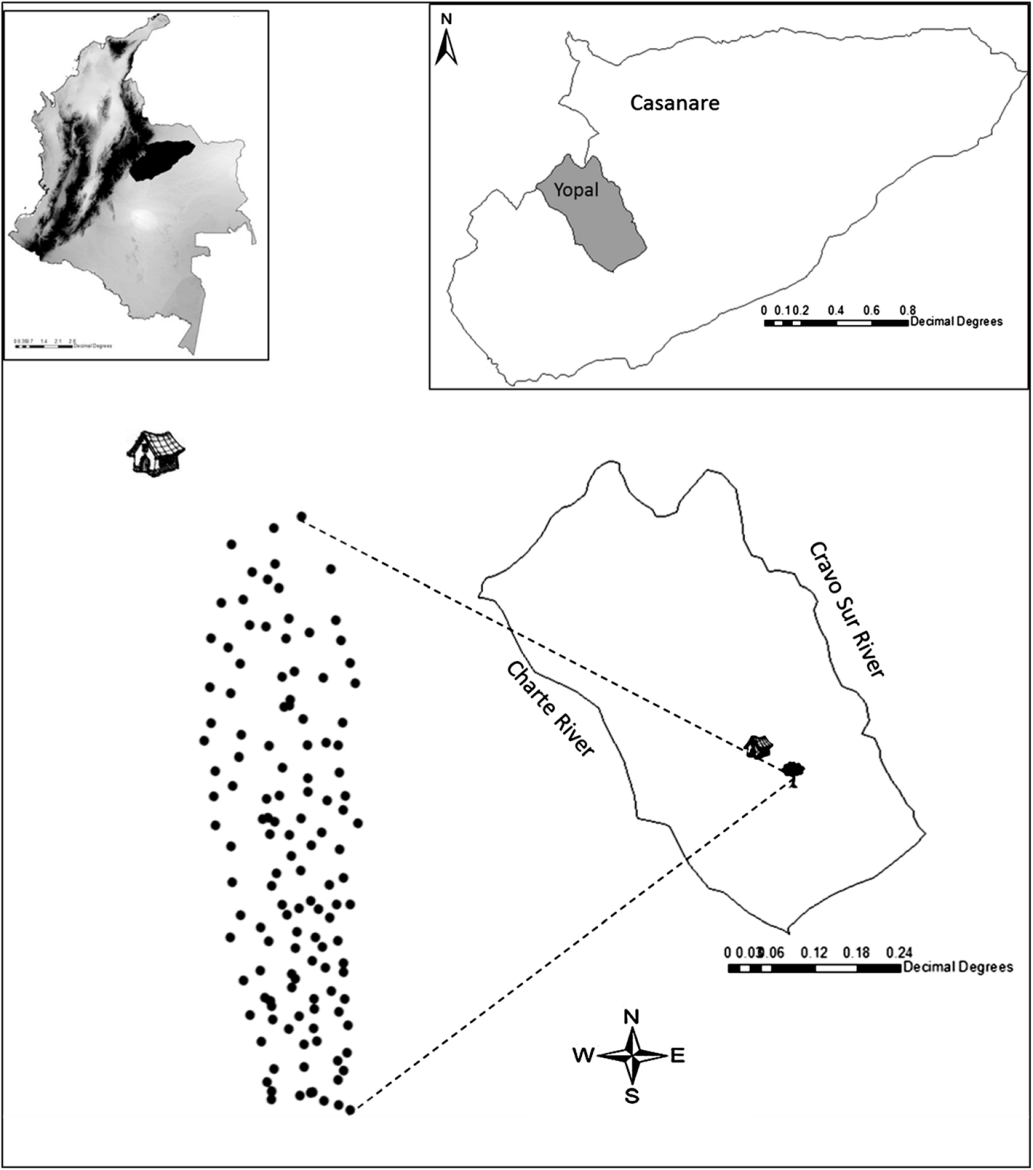
**Geographic location of the study area in Colombia.** The tree shows the location of the Corinto farm, Corregimiento Morichal in Yopal Casanare, and the points represent each of the individual palm trees sampled and their respective spatial distribution in the forest.

Study area: The sampling process was carried out between June and July 2012 in a 14-hectare natural *A. butyracea* forest that is located on the Corinto farm of the Corregimiento Morichal, Yopal municipality, Casanare, Colombia (5°10′50″North and 72°16′38″West). Gallery forests with different degrees of disturbance were observed in the study area. Disturbance mainly included cleared land for raising crops (corn, yucca and banana trees) and developing land for commercial purposes (grasslands for domestic livestock). Fragments of forests with palm trees of *Attalea butyracea* can be observed in this grassland landscape. Inside and close to the borders of the gallery forests wild fauna including opossums, bats, primates and anteater were observed. According to Romero et al. [36], this site corresponds to a dense high floodplain forest in the Cravo Sur River at 210 meters above sea level (m.a.s.l.) (Figure 1).

This area has a mean annual temperature of 24 ± 8°C and a mean annual precipitation of 2000–4000 millimeters, with a marked seasonality of unimodal regime. It also presents a period of high rain, which extends from April to October with a mean precipitation of 289.9 mm, and a period of low rain with a mean precipitation of 59.02 mm during the five remaining months. The sampling site is located in areas with evidence of *T. cruzi* transmission to humans and with houses near the native palm tree forests [37,38].

Selection of palm trees to sample: To determine the density of palm trees inside the forest ten plots randomly chosen of 0.25 hectares were delimited and all palm trees inside the plot were individually counted. Palm trees were identified following morphologically criteria establish by Galeano and Bernal [33] and confirmed by specialists from Universidad Nacional de Colombia-Bogotá.

Two groups of palm trees were sampled in the study. The first group of 150 palm trees was sampled only in the crown and included both subadults (individuals with the stem covered by old leaves from ground level to the crown and without evidence of reproduction) and adults (individuals with several leaves, visible stem and evidence of reproduction) palm trees [39]. The second group of plants include 40 adult palm trees with similar physiognomy (crown size, number of leaves, palm tree height, diameter at breast height (DBH), reproductive status) that were sampled in the base, mid-zone and crown, in order to determine differences in *R. prolixus* populations between the three zones.

To establish the plant to be sampled the following methodology was followed in both groups: the palm tree forest was delimited with a GPS (Garmin Etrex 10); then, 190 sampling points were randomly selected in the entire map, covering the entire forest area. Using the central point and closest neighbor technique reported by Gentry [40], the palm tree that was closest to each of the points marked with the GPS was sampled. For each sampled plant, the reproductive status (bract, inflorescence and fruit) and the phenotypic characteristics (height, diameter at breast height, number of leaves and crown size) were registered [31,32]. The height of the palm tree was measured from the stem’s base to the crown, the number of leaves were counted manually by climbing to the crown with metallic stairs, harnesses and safety belts.

In all cases the Euclidian distances from the edge of the forest to the sampled palm tree was determined using the ArcGis® 10.1 Software, using the corresponding geographic coordinates.

Sampling: The sampling process was carried out using live bait traps [19]. A first sampling of 150 palm trees with different physiognomy was carried out by installing one trap per palm tree crown every night. A second sampling of 40 palm trees with a similar number of leaves was carried out by simultaneously sampling three sites of their crown, as follows: the axillary part of the spirally arranged growing leaves with accumulation of dry biomass (crown base), the zone where the open and enclosed inflorescences start to branch (mid-zone) and the upper part looking to the apical meristem where new leaves are starting to grow and the old leaves are retained (crown). In each of these sites, a trap was installed for two consecutive nights.

In all cases, the traps were installed from 6:00 p.m. to 6:00 a.m. The traps were removed after 6:00 hours by climbing to the palm tree using metallic stairs and climbing equipment with harnesses and safety belts. The collected specimens were registered in a database for individual palm trees and for developmental stage, with nymphs from first to fifth instar (N1-N5) and adults. The captured specimens were transported in properly labeled bottles to the Centro de Investigaciones en Microbiología y Parasitología Tropical (CIMPAT) in Universidad de los Andes-Bogotá.

Identification of *R. prolixus*: The taxonomic identification of all individuals collected was made following the morphological criteria of the taxonomic keys of Lent and Wygodzinsky [3] and Carcavallo and Tonn [41].

Given the difficulty in defining the taxonomic status of *R. prolixus* due to the similarity of the morphological characteristics with the sympatric species *R. robustus* [3,41], the previously taxonomic identification was confirmed by molecular methods. For this analysis, an individual was randomly selected from the adults and fifth instar nymphs for each infested palm tree. In total, 169 individuals were selected. These individuals were macerated in guanidine chloride with 6 M EDTA and kept at room temperature for 24 h; then, DNA was extracted using the phenol-chloroform method [42]. A subsample of 15 individuals was selected from all 169 individuals whose DNA was extracted. From these 15, a 682-bp DNA fragment from region D2 of the *cytb* gene was amplified using primers cytb7432F 5′-GGACG(AT)GG(AT)ATTTATTATGGATC and cytb7433R 5′-GC(AT)CCAATTCA(AG)GTTA(AG)TAA. The reaction was carried out using the same conditions reported by Fitzpatrick et al. [43].

The purification of PCR products was carried out by using the Kit Wizard® SV from Promega. Then, the DNA concentration was quantified using a NanoDrop spectrophotometer, and 10-μL aliquots were selected for sequencing. The sequencing was carried out with the dideoxy-terminal method using both primers. The sequences were edited in MEGA 5.0 and analyzed by the NCBI BLAST to find the similarity percentage within the databases by using the default parameters.

Microclimatic variables: To evaluate the microclimatic behavior within the crowns, a *Data Logger* Hobo U23-001 was installed in one of the palm trees from the second sampling group for each of the three sampling sites. The temperature and relative humidity recordings were made at hourly intervals for 42 days. Then, the average and deviations of the logs of these variables were calculated for each hour to determine their behavior on the same day.

Entomological indices and natural infection rate: With the information collected for each sampled palm tree, the colonization, clustering, infestation and density indices were calculated according to Suarez-Dávalos et al. [29]. Following the methodology used by Ramírez et al. [44], the molecular detection of *T. cruzi* was carried out for all 169 insects whose DNA was extracted.

Statistical analysis: To check assumptions of data normality, the Shapiro-Wilk and Levene tests were performed to observe homoscedasticity. Simple and multiple regressions were made in the cases where the influence of the physiognomy of the palm trees on the *R. prolixus* relative density needed to be assessed. In this case, the number of leaves was treated as independent variables, given that it did not show correlation (Spearman’s p > 0.05). The Kruskal-Wallis test was carried out to evaluate statistically significant differences in *R. prolixus* densities according to the reproductive status of the palm tree and the microsite sampled in the same crown. The behavior of the developmental stage density per palm tree crown microsite was analyzed using Dunn’s multiple comparison tests.

A regression analysis of the relative population density and the developmental stage density with respect to the palm tree height and the distance from the edge of the forest was carried out. Additionally, the population disposition pattern in the forest was determined with an interpolation analysis, using the number of individuals collected and the geographic coordinates of the sampled palm trees and by applying the *Kriging* function (Euclidian distance) in the ArcGis 10.1 software.

The statistical concept was established under a significance level of 95% (p < 0.05), and GraphPad Prism 5.0 and Statistical Product and Service Solutions (SPSS) 17.0 software were used to perform the appropriate statistical analysis and graphics.

## Results

All captured individuals corresponded to *R. prolixus*, based on the morphological characteristics and on sequencing results (GenBank accession numbers: KP126725 to KP 126734), with 100% identity. From the 150 palm trees of the first sampling, 1339 insects were collected, with an average of 10.36 individuals per palm tree and ranging from 0 to 134 individuals. On the second sampling of 40 palm trees, 1071 insects were collected, with an average of 26.77 individuals per palm tree and ranging from 4 to 91 individuals (Table 1). In total, 313 first instar, 693 second instar, 622 third instar, 284 fourth instar, 287 fifth instar and 211 adults were collected (Table 2).

**Table 1:** **Infestation index and number of adults/nymphs of** ***Rhodnius prolixus*** **collected in the two samplings**

**Sampling**	**Sites sampled in the crown**	**Number of palm trees sampled**	**Number of palm trees infested (%)**	**Number of adults/nymphs collected**
First sampling	Crown	150	133 (88.66)	83/1256
Second sampling	Base	40	39 (97.5)	17/379
	Mid-zone	40	38 (95)	23/410
	Crown	40	35 (87.5)	88/154

**Table 2:** **Number of** ***Rhodnius prolixus*** **discriminated by instar and collected during the first and second sampling**

**Sampling (Number of palm trees sampled)**	**Instar**						**Total**
	**N1**	**N2**	**N3**	**N4**	**N5**	**ADULT**	
First sampling (150)	165 ± 0	405 ± 6	386 ± 4	162 ± 3	138 ± 2	83 ± 1	1339
Second sampling (40)	148 ± 2.6	288 ± 3.4	236 ± 3	122 ± 2.4	149 ± 2	128 ± 2	1071

**Figure 2:**
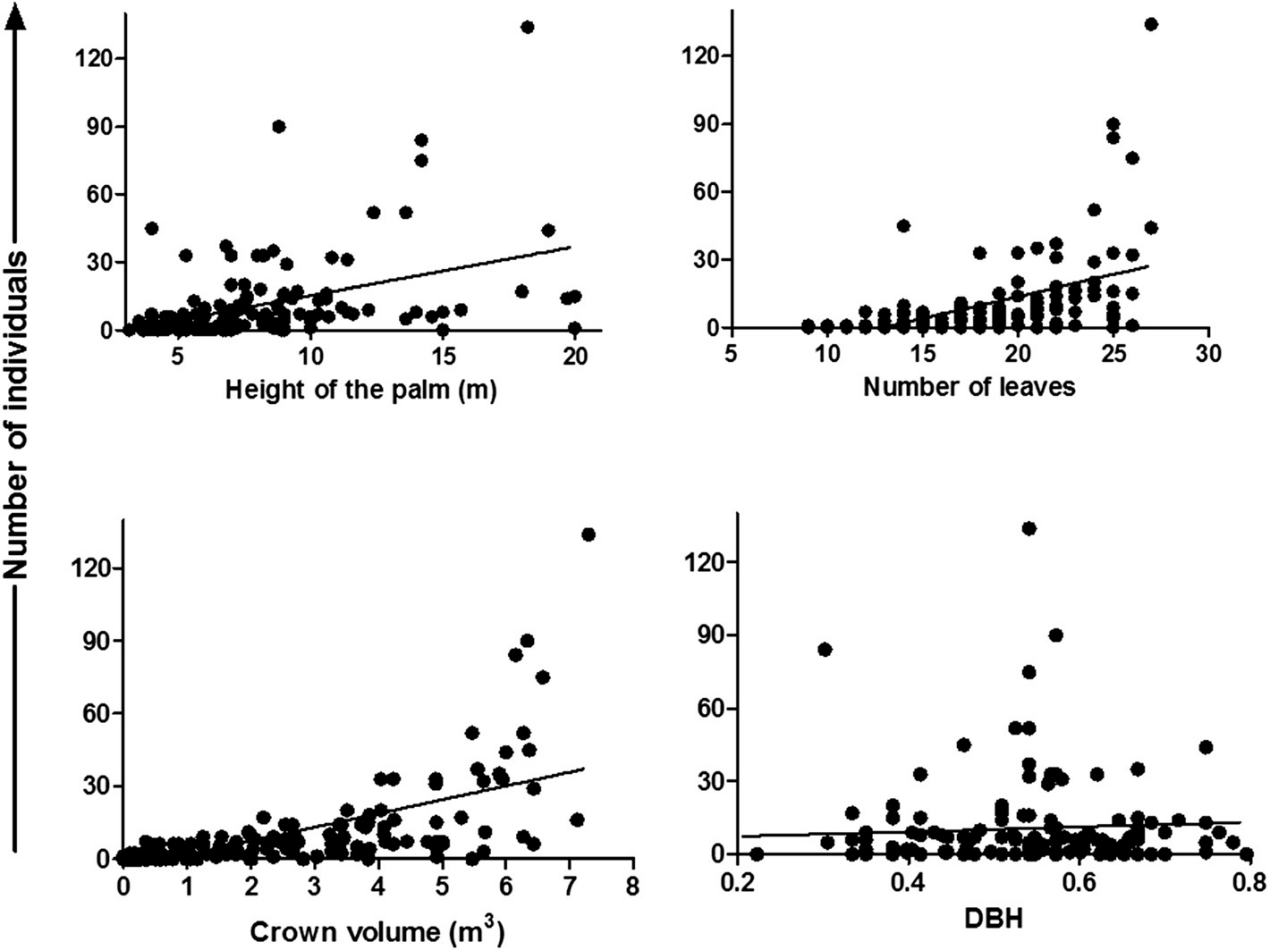
**Phenotypic characteristics of** ***Attalea butyracea*** **and population abundance of** ***Rhodnius prolixus.*** Association between phenotypic characteristics of *Attalea butyracea* palm trees and population abundance of *Rhodnius prolixus* (n = 150 palm trees). DBH, diameter at breast height.

Relative density of *R. prolixus* according to *A. butyracea* physiognomy: The 14-hectare natural forest of *A. butyracea* has an estimated density of 1265 palm trees/hectare. According to the regression analysis for the data of the 150 palm trees of the first sampling, the relative density of *R. prolixus* in *A. butyracea* natural forests is positively associated with the palm crown size (p < 0.0001; r^2^ = 0.41), number of leaves (p < 0.0001; r^2^ = 0.23) and palm tree height (p < 0.0001; r^2^ = 0.193). Palm trees with heights ranging from 2 to 20 meters were sampled and triatomines were collected in palm trees up to 2.9 meters. *R. prolixus* were also collected from palm trees with a number of leaves in the crown ranging from nine to 27 leaves. Conversely, the DBH of the palm tree has no influence on the population relative density (p = 0.25; r^2^ = 0.0088) (Figure 2). A multiple regression analysis carried out with the variables that presented an association with the *R. prolixus* relative density showed a high significance (p = 0.000; r^2^ = 0.64). This result confirms that the palm tree crown structure affects the relative population density of *R. prolixus*.

**Figure 3:**
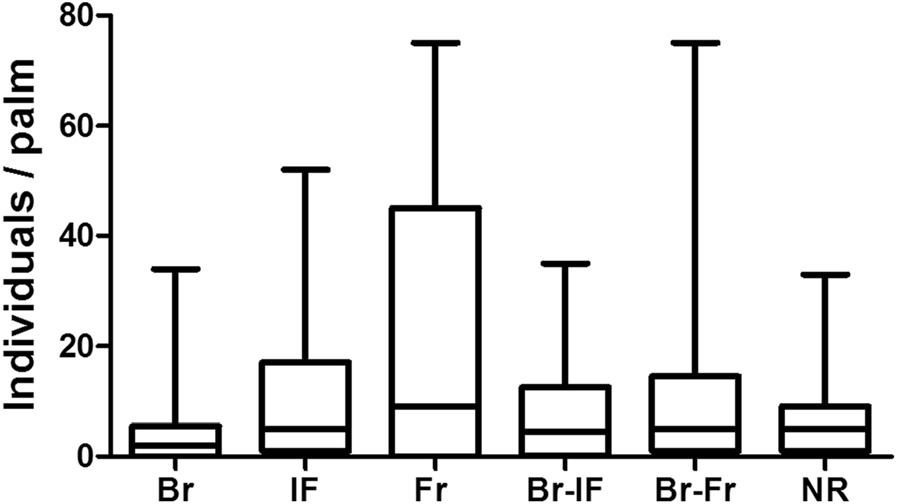
**Population density (nymphs and adults) of** ***Rhodnius prolixus*** **according to the reproductive status of** ***Attalea butyracea*** **palm trees (n = 150 palm trees).** Br, bract; IF, Inflorescence; Fr, Fruit; Br-IF, Bract and inflorescence; Br-Fr, bract and fruit; and NR, non-reproductive.

Population relative density of *R. prolixus* according to the reproductive status of *A. butyracea*: No statistically significant differences between the relative population density (adults and nymphs) of *R. prolixus* and the reproductive status of the palm tree were found (Kruskal-Wallis, p = 0.53) (Figure 3).

**Figure 4:**
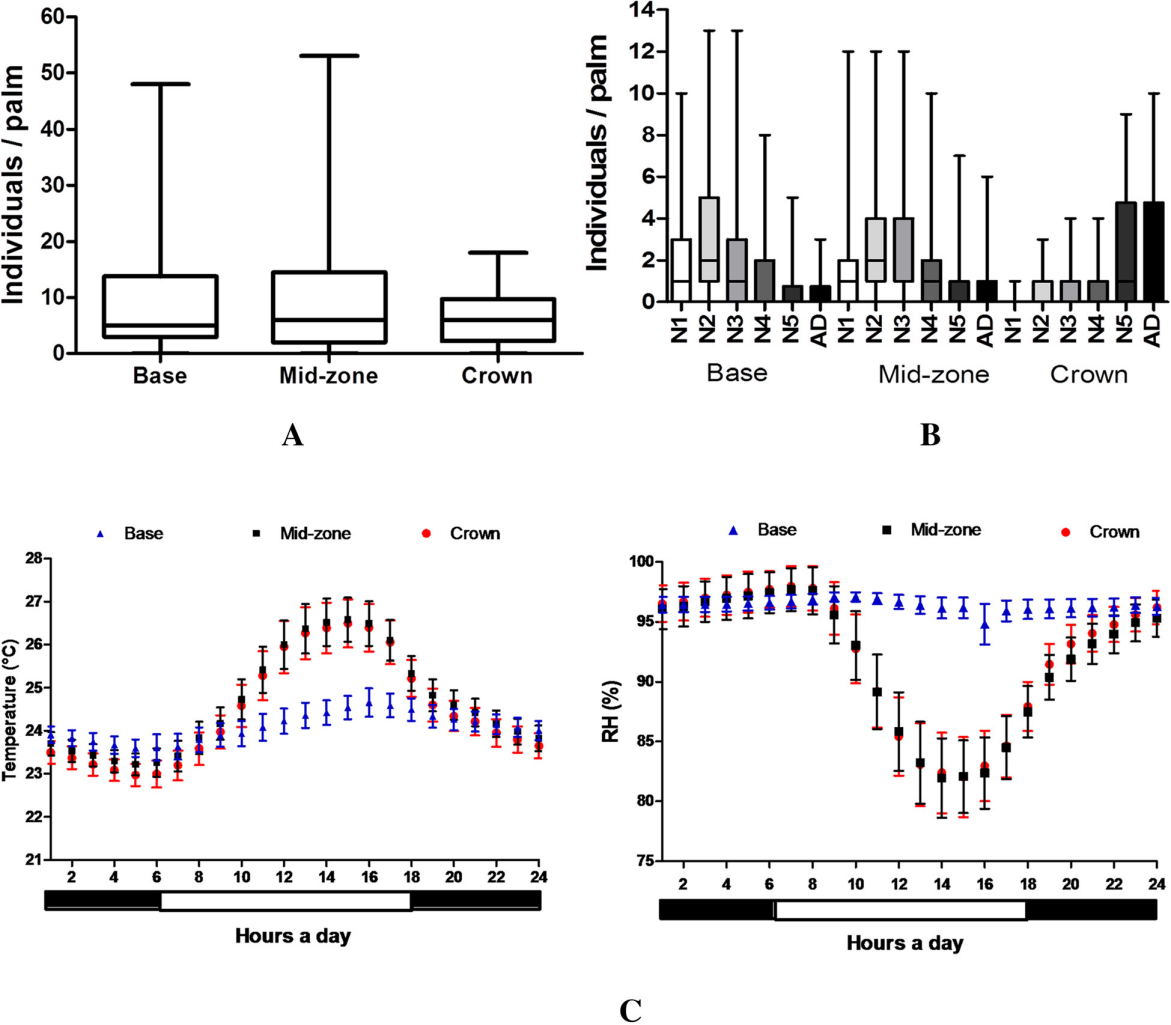
**Features of palm trees with similarities in their physiognomic structure (n = 40 palm trees). A)** Population density (nymphs and adults) of *Rhodnius prolixus* by the microhabitat sampled in *Attalea butyracea* palm tree crowns. **B)** Stratification density of the developmental stages of *Rhodnius prolixus* in crowns of *Attalea butyracea* palm trees. **C)** Daily variations of the temperature and relative humidity in the three sampling sites on the crown of *Attalea butyracea* palm tree.

Population relative density and age structure of *R. prolixus* in the palm tree crown: No statistically significant differences between the *R. prolixus* relative population density were found according to the sampling site in the crown (Kruskal-Wallis, p = 0.54) (Figure 4A). However, the N1, N2 and N3 relative densities were significantly higher in the base and mid-zone of the crown (Dunn, p = 0.001), while the N5 and adult relative densities were significantly higher than the rest of the developmental stages in the crown (Dunn, p = 0.0001) (Figure 4B).

Temperature and relative humidity on palm tree crown: According to the data of the environmental variables, the mid-zone and the crown of the palm tree showed a lower relative humidity of nearly 12% and an increase in temperature of approximately 3°C during the day. These data indicate that the crown’s base has an increased microclimatic stability, compared to the other sites (Figure 4C).

**Figure 5:**
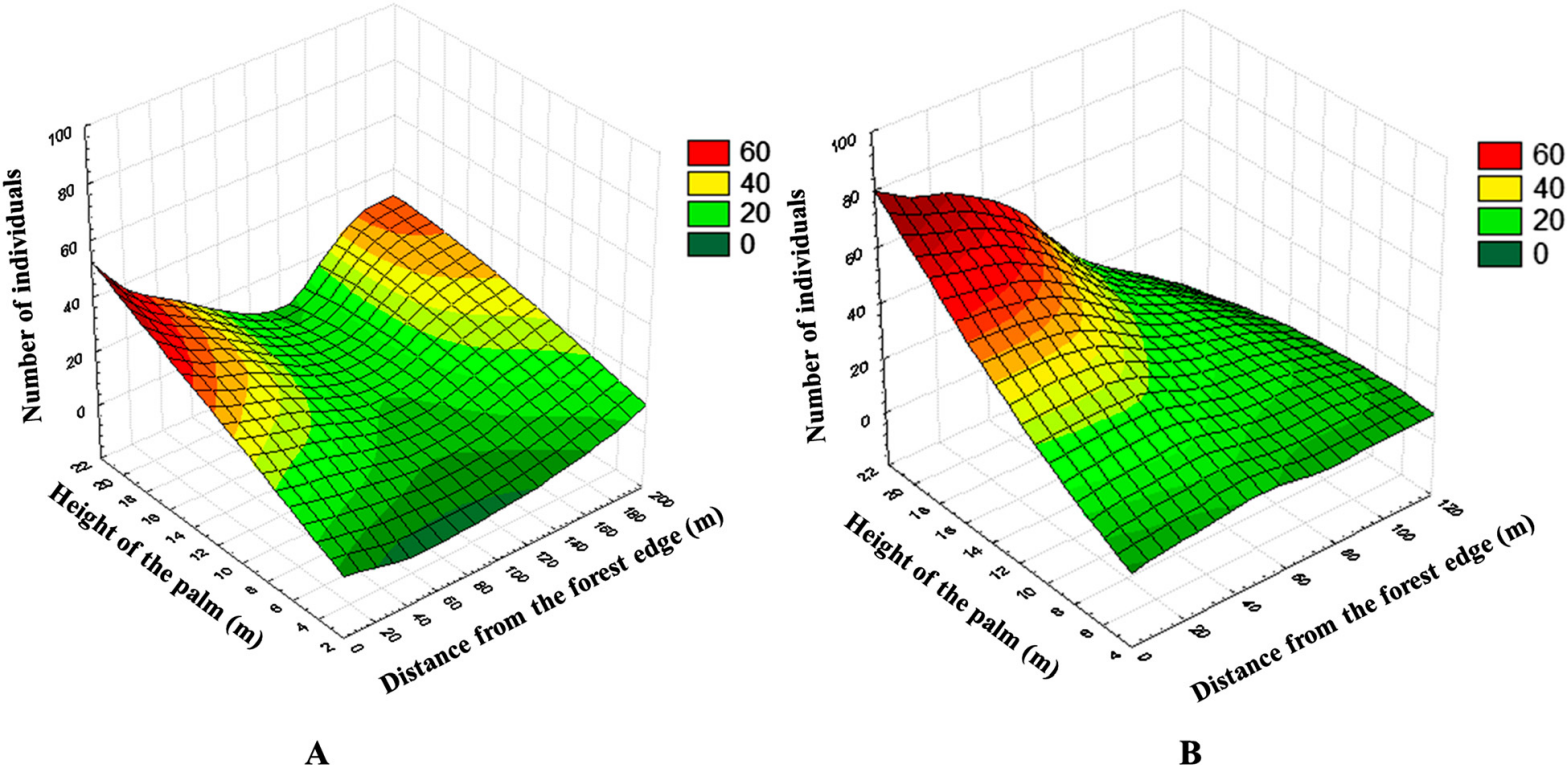
**Vertical and horizontal stratification of the population abundance of** ***Rhodnius prolixus*** **in an** ***Attalea butyracea*** **forest. A)** Abundance of *R. prolixus* in palm trees with a structural variation in their physiognomy (n = 150 palm trees). **B)** Abundance of *R. prolixus* in palm trees with a structural similarity in their physiognomy (n = 40 palm trees).

*R. prolixus* population distribution in palm tree forest: According to the regression analysis carried out with the data of the first sampling, a significant influence of the distance (p = 0.003; r^2^ = 0.107) and the palm tree height (p = 0.0001; r^2^ = 0.205) was found. This result indicates a horizontal and vertical stratification of the population density in the forest (Figure 5A). This same analysis, which was carried out on the relative densities found in the 40 palm trees of the second sampling, also showed the same pattern but with a greater significance level for the height of the palm tree (p = 0.00001; r^2^ = 0.33) (Figure 5B).

This trend of horizontal distribution was also found for some of the developmental stages, such as N1 (p = 0.0025; r^2^ = 0.06), N2 (p = 0.0032; r^2^ = 0.05), N3 (p = 0.003; r^2^ = 0.08), N4 (p = 0.0029; r^2^ = 0.058) and adults (p = 0.019; r^2^ = 0.036), but with low determination coefficients. A stratification tendency in all stages was also observed as the height of the palm trees increased (p = 0.0001). However, the interaction level was different for each one of the instars, with N3 (r^2^ = 0.19) and N4 (r^2^ = 0.22) showing the highest association and N1 (r^2^ = 0.10), N2 (r^2^ = 0.13), N5 (r^2^ = 0.12) and adults (r^2^ = 0.13) showing much lower r^2^ values.

**Figure 6:**
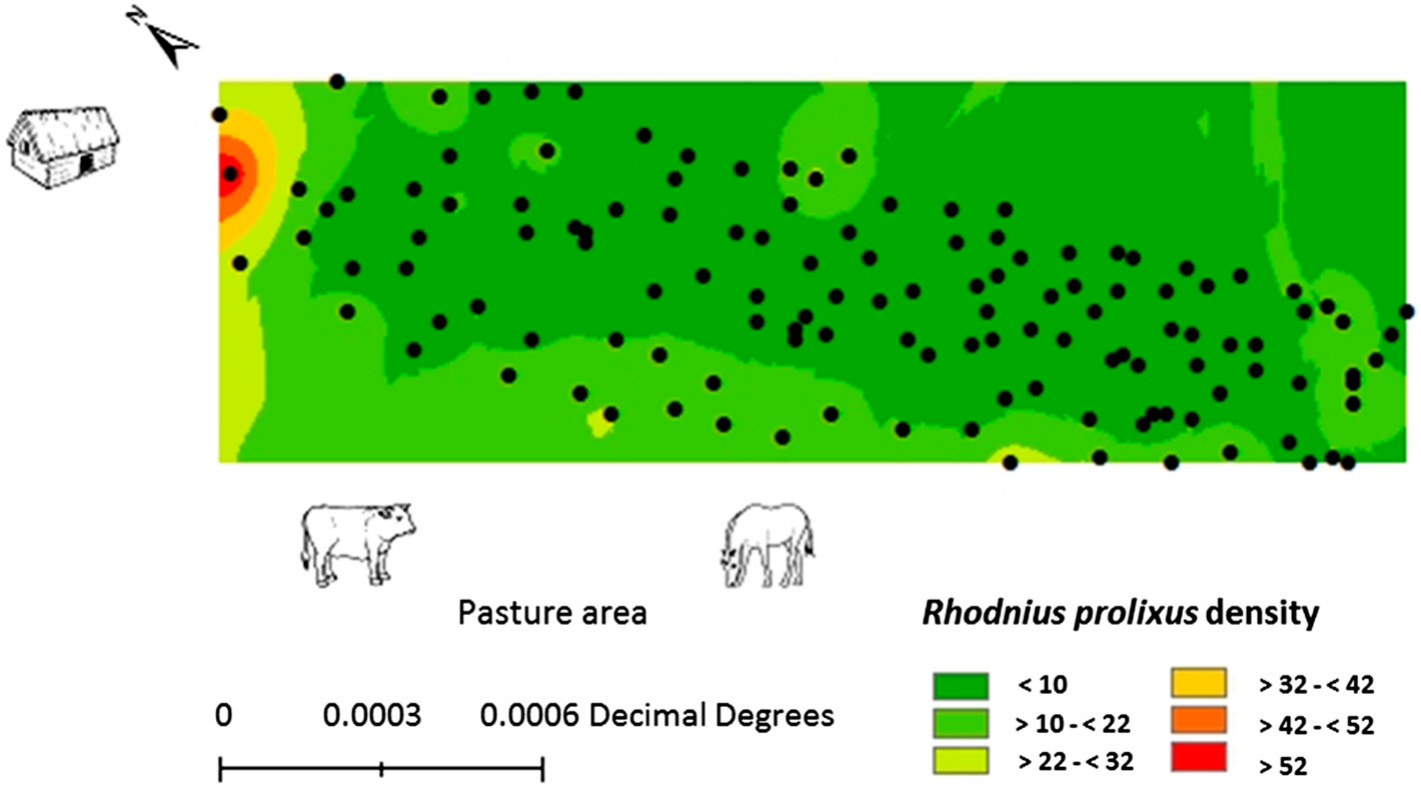
**Interpolation of the abundance of** ***Rhodnius prolixus*** **in the sampled forest (n = 150 palm trees).** The rectangle represents the forest area and its proximity to houses and intervened pasture areas. Each point represents the geographical position of each of the sampled palm trees.

Conversely, the interpolation analysis of the relative densities collected showed that the highest *R. prolixus* population density was distributed towards the forest area which is closer to the housing zone. However, the analysis also showed that there were high densities towards the forest area that was near the pasture-intended areas (Figure 6). Together, these results indicate that *R. prolixus* is present throughout the entire forest, with a higher population density towards the upper strata and the edge of the forest, specifically towards the peridomestic environments and anthropic areas.

Entomological indices: A density index of 12.6 individuals per palm tree, clustering index of 14.9 individuals per palm tree, infestation index of 88.9% and colonization index of 98.7% were registered. The infection index that was determined by the *T. cruzi* DNA detection was 85.2% (144 positive insects out of 169 analyzed).

## Discussion

The methodology used in this study has the advantage that it avoids the cutting and the dissection of the palm trees [19]. The live bait traps used here are easy to transport and able to collect triatomines from different stages and species [19]. In addition, the efficacy of the live bait trap to collect triatomines in *A. butyracea* was previously highlighted [19]. Finally, the size of the trap allowed us to sample effectively different parts in the crown of the palm trees. However, the reduced number of traps used in the study has a disadvantage that we can only present results as relative densities of insects. Eggs and exuvia from the insects are also not able to be collected with this methodology.

Morphological identification of all collected insects and the molecular confirmation of some individuals allowed us to confirm the presence of *R. prolixus* in the palm tree forest sampled. However, due to the presence of *R. robustus* in Colombia [25], the difficulty to identify morphologically the immature stages of *Rhodnius* [3] and budget constraints for molecular confirmation of all collected insects, we leave open a small possibility that some nymph instars collected could be *R. robustus*. A recent publication leaves open the possibility to use the external female genitalia as a tool for the future morphological identification of *Rhodnius* species [45].

The high influence of the structural physiognomy of the palm tree on the population relative density of *R. prolixus* (Figure 2) shows a strong association of the vector with the structural features of the palm tree. These results have also been found by Pizarro and Romaña [25], Gaunt and Miles [22], Teixeira et al. [14] and Abad-Franch et al. [15]. These studies have also allowed the classification of palm trees as an important ecotope for the maintenance of colonies of different *Rhodnius* species.

The result that the highest level of interaction on *R. prolixus* relative population density has been determined by the crown size shows a decisive role of this variable over the microhabitat possibilities for the establishment of the vector. This feature is because *A. butyracea* has leaf abscission patterns that generate constant necromass accumulation processes in their crowns. This characteristic promotes the increase on crown volume and, consequently, provides the ideal habitat conditions for triatomines [46]. Moreover, there is a correlation between the reproductive events of *A. butyracea* individuals and leaf formation, which has a secondary effect not only on the size of their crown but also on their height [31]. This fact could explain the high significance found between the palm tree height and the number of leaves with the relative population density of *R. prolixus*. This result is similar to what Abad-Franch et al. [47] has reported for triatomine populations sampled on the palms of the genus *Attalea* in the Amazon.

Alternatively, palm trees are an important source of energy transformation in tropical forests due to their high production of flowers and fruits, marked phenophase synchrony and reproductive events that are accompanied by an increase in their thermogenic condition [32,33]. These biological characteristics favor the supply of much more stable microclimate conditions for floral visitors during reproductive periods [31,32] and also the arrival and nesting of vertebrates, which offer food for triatomines [34,48]. However, our results show that the reproductive status of *A. butyracea* does not significantly favor the population density of *R. prolixus* on their crowns (Figure 3). Therefore, for this vector, the colonization and establishment process does not depend on the reproductive condition of the palm tree but instead depends on its structural physiognomy and anthropic impact in the forest as has been shown for other triatomine species [49].

The homogeneous distribution of population relative densities found (Figure 4A) and the spatial stratification of the developmental stages in the crown (Figure 4B) show that *R. prolixus* is effectively occupying all the microsites offered by the ecotope. This result possibly occurs as an efficient strategy of habitat exploitation because it has been found that *R. prolixus* has a high sensitivity to environmental changes to which it adapts easily [48,50]. Some physiological adaptive responses of triatomines to adverse environments, such as nymphal period length variation, molting rates, hatching eggs, adult longevity, egg-laying events and starvation periods, which generate variation in the representativeness of developmental stages in spatial units [13,41,48,51], could be influencing the presence of the stages found within the palm tree crown (Figure 4B).

Considering that the developmental stages show different tolerance ranges to both the environmental and food conditions [52,53], the variation of their presence according to the sites sampled in the palm tree (Figure 4B) could also be influenced by the variation in the temperature and relative humidity measured in one palm tree sampled in its mid-zone and crown (Figure 4C). In addition, the higher density of individuals at the higher stages in the crown of the palm tree could be due to a better adaptation to environmental changes in those stages [53]. In this sense, *R. prolixus* could be rearranging its age structure according to the conditions of the microhabitat measured here. This feature allows the insect to efficiently occupy the microsites offered by the crown of the palm trees and to ensure the spatial stability of the population.

Alternatively, the highest relative population density observed in relation to the palm tree height (Figure 5) could be explained by the offer of better life conditions for the development of colonies given that these palms represent the most mature stratum of the palm grove and therefore have achieved not only a high biomass accumulation in their crowns but also the establishment of more stable secondary interactions with vertebrates, given their multiple reproductive events [31,32].

The highest population relative density found at a shorter distance from the forest edge (Figure 5), especially towards intervened areas for pastures and peridomestic environments (Figure 6), could be explained by a higher palm tree density and better food availability in those sites. These conditions may determine the vector’s population distribution. However, given the high density of palm trees in the forest (1265 palms/hectare), the observed spatial distribution would be determined by a condition of greater food supply and not of microhabitats.

This fact could be explained by an increased supply of food resources towards intervened areas, which is guaranteed by the constant influx of opportunistic vertebrates as a result of a possible edge effect [54] and the offer of food by cattle and horses in pasture areas and from domestic animals in peridomestic areas (Figure 6). This event would also agree with Diotaiuti and Dias [55], who argue that the offer and the type of food resource can affect the spatial distribution of triatomines both at population level and at developmental stage. Additionally, it agrees with the reports from Suarez-Dávalos et al. [29], who found that the triatomine population density increases near houses.

In this regard, studies on the population dynamics of mammals such as the didelphids, bats, rodents and primates associated with these ecosystems (which also appear as the main reservoir groups of *T. cruzi* [56-58]) are relevant to understanding the scope of the interaction between the vertebrates and triatomines on the vector’s population micro-distribution and the dynamics of the vector’s wild cycle. The wild reservoirs condition the food supply in the wild ecosystems, and the sympatric nature of the host-parasite encounter affects the success of the infection [56].

In terms of developmental stages, the nymphs have a restricted dispersal to new sites, while adults have high dispersal ability [42,59]. Our results reflect this situation in the vertical and horizontal stratification found for the entire studied population (Figures 5 and 6). This feature is also reflected when each of the development stages were analyzed, where the majority have the same population pattern. The low significance of adult stratification can be explained by their dispersion ability [59], which ensures a constant and uniform distribution of individuals seeking colonization sites within the forest.

The high infection and colonization rates found reflect a high capacity for dispersal and colonization in the studied forest. The density and clustering indices recorded show a population spatial stability of the vector, which is guaranteed by an efficient niche colonization strategy. These results together indicate a strong association of the vector with the palm tree groves, which guarantees high population densities and is consistent with the findings in other studies with *R. prolixus* [20,24] and other *Rhodnius* species [29,47,60].

Conversely, the registered rate of infection by *T. cruzi* (85.2%) was higher in comparison with other studies with triatomines in palm trees [14,15,17,20,21,25,29] but comparable to those reported in other endemic areas [61]. This fact, coupled with the efficient dispersal ability of *R. prolixus* [59], the temporal stability of the population density in these forests [24] and the population distribution found (Figure 6), represents a high risk factor for the transmission of the parasite to the people surrounding these wild environments. This event is also favored by the diversity of palm tree landscapes that are immersed in the peridomestic areas on the eastern plains of Colombia, which creates ideal conditions for the spatial-temporal establishment of *R. prolixus*.

## Conclusions

The presence and population relative density of *R. prolixus* in wild palm tree groves is determined by the structural characteristics of the crowns and not by the reproductive condition of the palm tree, where those presenting the greatest volume and number of leaves recorded higher population densities of *R. prolixus*. The microclimatic conditions in the crown of palm trees in turn affect the spatial organization of the age structure of *R. prolixus*, as the higher stages are distributed in crown sites where there is greater variation of the environmental conditions.

According to the high values of density, colonization, infestation and spatial distribution of *R. prolixus*, together with the high rate of infection with *T. cruzi* registered, the presence of forest units and palm trees near homes constitutes a risk factor for the migration of infected *R. prolixus* towards domestic environments and its consequent epidemiological risk of transmission of *T. cruzi* to people from wild environments.

## Abbreviations

PCR: Polymerase chain reaction
DNA: Deoxyribonucleic acid
BLAST: Basic local alignment search tool
NCBI: National center for biotechnology information
DBH: Diameter at breast height
Br: Bract
IF: Inflorescence
Fr: Fruit
Br-IF: Bract and inflorescence
Br-Fr: Bract and fruit and
NR: Non-reproductive
N1: First instar nymphs
N2: Second instar nymphs
N3: Third instar nymphs
N4: Fourth instar nymphs
N5: Fifth instar nymphs and
AD: Adult
